# Molecular basis for DNA strand displacement by NHEJ repair polymerases

**DOI:** 10.1093/nar/gkv965

**Published:** 2015-09-23

**Authors:** Edward J. Bartlett, Nigel C. Brissett, Przemyslaw Plocinski, Tom Carlberg, Aidan J. Doherty

**Affiliations:** Genome Damage and Stability Centre, School of Life Sciences, University of Sussex, Brighton, BN1 9RQ, UK

## Abstract

The non-homologous end-joining (NHEJ) pathway repairs DNA double-strand breaks (DSBs) in all domains of life. Archaea and bacteria utilize a conserved set of multifunctional proteins in a pathway termed Archaeo-Prokaryotic (AP) NHEJ that facilitates DSB repair. Archaeal NHEJ polymerases (Pol) are capable of strand displacement synthesis, whilst filling DNA gaps or partially annealed DNA ends, which can give rise to unligatable intermediates. However, an associated NHEJ phosphoesterase (PE) resects these products to ensure that efficient ligation occurs. Here, we describe the crystal structures of these archaeal (*Methanocella paludicola*) NHEJ nuclease and polymerase enzymes, demonstrating their strict structural conservation with their bacterial NHEJ counterparts. Structural analysis, in conjunction with biochemical studies, has uncovered the molecular basis for DNA strand displacement synthesis in AP-NHEJ, revealing the mechanisms that enable Pol and PE to displace annealed bases to facilitate their respective roles in DSB repair.

## INTRODUCTION

Cellular life must overcome a vast array of undesirable genomic alterations in order to replicate and divide. One of the most catastrophic types of DNA damage are double-strand breaks (DSBs) (1). There are two evolutionarily conserved mechanisms that cells utilize to repair DSBs; homologous recombination (HR) and non homologous end-joining (NHEJ). The NHEJ pathway allows for the direct ligation of broken DNA termini and is also capable of reconfiguring incompatible ends, whilst the HR pathway replicates new DNA using a homologous template. The template free nature of NHEJ allows this process to be potentially active throughout the cell cycle of any organism that possesses the necessary genes, and is the mechanism that is most frequently used to repair DSBs in higher eukaryotic cells (2).

The canonical mechanism of NHEJ in mammalian cells is initiated by the binding of the Ku70/80 heterodimer to the broken DNA ends, along with the DNA-dependent protein kinase catalytic subunit (DNA–PKcs), reviewed in references (3) and (4). Ku70/80 and DNA–PKcs encourage a synapsis of the DNA termini and recruitment of the remaining core NHEJ factors; Ligase 4 (Lig4), X-ray repair cross-complementing protein 4 (XRCC4) and XRCC4-like factor (XLF). Further processing of complex DSBs requires additional proteins, including polynucleotide kinase phosphatase (PNK) that catalyses removal of the 3′-phosphate (3′-P) and 5′-hydroxyl groups (5), and Artemis which provides endonucleolytic and exonucleolytic activities (6,7). DNA polymerases μ and λ possess unique capabilities that enable them to incorporate nucleotides to blunt dsDNA ends and fill ssDNA gaps (8). Together, these repair enzymes are able to rectify severe genetic alterations that potentially comprise a large volume of different combinations.

NHEJ is one of the most fundamental mechanisms for sustaining genome stability and first evolved in bacteria (9). Recently, we described an analogous system in the so-called third domain of life, archaea. The archaeal and bacterial NHEJ systems studied to date are highly homologous and therefore this archetypal pathway has been termed archaeo-prokaryotic (AP) NHEJ (10). The AP–NHEJ repair complex is primarily composed of a Ku homodimer, DNA ligase (Lig), DNA polymerase (Pol) and a phosphoesterase (PE) (10–14). In mycobacteria, and many other bacterial species, the Lig, Pol and PE components are fused together to form a single, contiguous multifunctional protein called Ligase D (13). However, in many organisms these proteins are separate but co-expressed from a single operon. The *Mycobacterium tuberculosis* (*Mtu*) AP–NHEJ complex is the most extensively characterized system (13–16). Studies on NHEJ repair in *Mtu* and the archaeon *Methanocella paludicola* (*Mpa*) demonstrated that both species possess equivalent core AP–NHEJ components, with orthologous proteins possessing near identical substrate specificities (10,14). Surprisingly, the NHEJ repair process makes use of ribonucleotides during DNA repair, with all three enzymatic subunits having strict preference, or even necessities, for RNA (10,14-19). The NHEJ polymerase (PolDom/LigDPol) preferentially incorporates ribonucleotides into gaps or blunt dsDNA ends. The PE nuclease subunit requires the presence of a hydroxyl in the 2′ position (2′-OH) of the ribose moiety, which chemically differentiates RNA from DNA, in order to remove ribonucleosides. Whilst Lig requires a ribobase at the 3′ side of the nick to efficiently catalyse phosphodiester bond formation to seal the break back together (10,18).

We recently elucidated a role for PE in AP–NHEJ, demonstrating that it acts as as a regulator of the polymerase activity. We discovered that Pol has DNA strand displacement activity during RNA synthesis and that PE resects excessive incorporation to promote efficient break ligation (10). In this current study, we sought to extend our understanding of AP–NHEJ by comparing crystal structures of the archaeal *(Mpa)* Pol and PE with those of the established bacterial (*M. tuberculosis* and *P. aeruginosa*) DSB repair complexes (15,16,20,21). Examination of the structural features of *Mpa* PE, along with biochemical studies, identified that a conserved active site histidine is critical for disrupting annealed terminal 3′-nucleotides to allow catalytically competent positioning of the scissile phosphate. Furthermore, we report that the displacement synthesis activity of Pol is conserved in AP–NHEJ repair and is regulated by PE. As part of its normal synthesis cycle, Pol distorts the templating strand to incorporate the incoming base into the active site. However, this can lead to template dislocation and strand displacement when a downstream strand is present. Examination of the crystal structures of Pol reveals that these enzymes contain a prominent surface protrusion that plays a critical role in this displacement activity. We identify that a conserved arginine residue in this structure acts as a molecular wedge against the annealed interface of the downstream DNA and is critical for enabling displacement synthesis during polymerase extension that occurs during AP–NHEJ repair. We propose that this displacement activity is maintained by these enzymes in order to enhance their capacity to promote break synapsis by a process known as microhomology mediated end-joining (MMEJ).

## MATERIALS AND METHODS

### Crystallization of *Mpa* phosphoesterase and *Mpa* polymerase

Full-length *Mpa* PE was purified as described in (10). 0.7 μl of protein solution (at a concentration of 30 mg/ml) was set up in sitting drop experiment against 200 mM magnesium sulfate, 20% (w/v) PEG 3350 at a ratio of 1:1 next to a reservoir of 700 μl, and the drops were incubated at 12ºC. Typically, crystals appeared after 2–3 weeks and grew to full size by 4–5 weeks. A day prior to data collection, crystals were soaked in 10 mM sodium vanadate. Crystals were harvested and cryoprotected in reservoir buffer plus 17% (v/v) ethylene glycol before snap freezing in liquid nitrogen. The data sets for *Mpa* PE vanadate were collected at a synchrotron light source, Station IO3, Diamond (Oxfordshire, UK). The diffraction data were processed with SCALA (22) with additional processing by programs from the CCP4 suite (23). The statistics for data processing are summarized in Table 1.

**Table 1.** Data collection and refinement statistics for the *Mpa* PE crystal structure

**Data collection**	
Source	Station IO3, Diamond
Space group	P3_1_21
Unit cell dimensions	
a, b, c (Å)	57.07/57.07/105.04
α, β, γ	90.00/90.00/120.00
Wavelength (Å)	0.9763
Resolution (Å)	49.43–1.79
Total number of observations	95 174
Number of unique reflections	18 976
Overall I/(σI)^a^	16.4 (3.2)
Overall completeness (%)^a^	98.9 (99.9)
R_sym_ (%)^a,b^	0.05 (0.45)
Redundancy^a^	5.0 (4.9)

**Refinement**	
Resolution (Å)	49.42–1.79
Number of reflections	17 965
R_factor_ / R_free_^c,d^	0.1744/0.2045
Number of atoms	
Protein	1341
Water molecules	90
Mean B value (Å^2^)	37.5
Rmsds	
Bonds (Å)	0.020
Angles (°)	2.070
Ramachandran statistics	
Favoured regions (%)	94.61
Allowed regions (%)	4.79
Disallowed regions (%)	0.60
PDB ID	5 DMP

^a^Values for highest resolution shell (1.89–1.79Å) is in parentheses.

^b^*R*_sym_ = Σ|*I* – <*I*>| / Σ <*I*>, where *I* is the observed intensity.

^c^*R*_factor_ = Σ∥*F*o – |*F*c∥ / Σ|*F*o|, where *F*o and *F*c are the observed and calculated structure factor, respectively.

^d^*R*_free_ is equal to R factor for a randomly selected 5% subset of reflections not used in the refinement.

Full-length *Mpa* Pol was purified as described in (10). 0.7 μl of protein solution (with concentrations ranging from 2.5 to 7.65 mg/ml) was set up in a sitting drop experiment against 200 mM ammonium sulfate, 20% (w/v) PEG 3350 at a ratio of 1:1 next to a reservoir of 700 μl, and the drops were incubated at 12ºC. Typically, crystals grew to full size within 1–2 weeks. Crystals were harvested and cryoprotected in reservoir buffer plus 17% (v/v) ethylene glycol before snap freezing in liquid nitrogen. All data sets were collected at 100 K. Single wavelength diffraction data of *Mpa* Pol were collected in-house on a Rigaku Micromax 007-HF. The diffraction data were processed with SCALA (22) with additional processing by programs from the CCP4 suite (23). The statistics for data processing are summarized in Table 2.

**Table 2.** Data collection and refinement statistics for the *Mpa* Pol crystal structure

**Data collection**	
Source	Rigaku MicroMax 007-HF
Space group	P21
Unit cell dimensions	
a, b, c (Å)	44.42/60.55/59.41
α, β, γ	90.00/101.02/90.00
Wavelength (Å)	1.5418
Resolution (Å)	13.934–1.949
Total number of observations	77013
Number of unique reflections	22 140
Overall I/(σI)^a^	13.8 (4.9)
Overall completeness (%)^a^	97.4 (84.0)
R_sym_ (%)^a,b^	0.07 (0.20)
Redundancy^a^	3.5 (2.6)

**Refinement**	
Resolution (Å)	13.934–1.949
Number of reflections	20 996
R_factor_ / R_free_^c,d^	0.1478/0.1954
Number of atoms	
Protein	2429
Water molecules	288
Mean B value (Å^2^)	15.01
Rmsds	
Bonds (Å)	0.02
Angles (°)	1.82
Ramachandran statistics	
Favoured regions (%)	98.28
Allowed regions (%)	1.72
Disallowed regions (%)	0
PDB ID	5 DMU

^a^Values for highest resolution shell (2.05–1.95 Å) is in parentheses.

^b^*R*_sym_ = Σ|*I* – <*I*>| / Σ <*I*>, where *I* is the observed intensity.

^c^*R*_factor_ = Σ∥*F*o – |*F*c∥ / Σ|*F*o|, where *F*o and *F*c are the observed and calculated structure factor, respectively.

^d^*R*_free_ is equal to R factor for a randomly selected 5% subset of reflections not used in the refinement.

### Structure solution and refinement of *Mpa* PE and Pol

The structure of *Mpa* PE was determined by molecular replacement using the program PHASER (24). The crystallographic model of *Pseudomonas aeruginosa* PE (PDB: 3N9B) was used as a molecular replacement search model (21). Initial refinement was carried out against 95% of the data with REFMAC5 (25). The remaining 5%, which were randomly excluded from the full data set, was used for cross-validation by calculating the Rfree to follow the progress of the refinement. The same subset of reflections was used throughout the refinement. Each cycle of refinement was accompanied by manual rebuilding using the program COOT (26). The structure images were prepared with PyMol and CCP4mg (27,28).

The structure of *Mpa* Pol was determined by molecular replacement using the program PHASER (24). The crystallographic model of *Mtu* PolDom (PDB: 2IRU) was used as a molecular replacement search model. Initial refinement was carried out against 95% of the data with REFMAC5 (25). The remaining 5%, which were randomly excluded from the full data set, was used for cross-validation by calculating the Rfree to follow the progress of the refinement. The same subset of reflections was used throughout the refinement. Each cycle of refinement was accompanied by manual rebuilding using the program COOT (26). The structure images were prepared with CCP4mg (27).

### Protein alignments

The amino acid sequences of both the putative AP–NHEJ phosphoesterases and polymerases were gathered by BLAST search from Genbank (29,30). The sequences were aligned with ClustalW2 (31).

### Construction and purification of *Mtu* PolDom wt and mutants, *Mpa* Pol and *Mpa* PE wt and mutant

*Mtu* PolDom and *Mpa* PE mutants were generated by site-directed mutagenesis (QuickChange, Stratagene) of the overexpression plasmids for wild-type (wt) *Mtu* PolDom and *Mpa* PE, respectively (mutagenesis primers listed in Supplementary Table S1). The DNA constructs were then sequenced to verify accurate mutation and subsequently transformed into *E. coli* B834(DE3)pLysS. Purification of all *Mtu* PolDom proteins was performed as described in (20). Purification of the *Mpa* Pol and wt and H82A PE were performed as described in (10).

### RNA polymerization assays

RNA extension of DNA primers was performed as described in (10). Briefly, the incubation mixture contained 50 mM Tris-HCl (pH 7.5), 5 mM MnCl_2_, 30 nM Fluorescein labelled DNA, the indicated concentration of NTPs, and either wild-type *Mpa* Pol, *Mtu* PolDom or the indicated mutants, in a volume of 20 μl. After 60 min of incubation at 37ºC, reactions were stopped by adding Stop buffer (95% [v/v] formamide, 0.09% [w/v] xylene cyanol) and then boiled at 95ºC for 10 min. The DNA products were separated by electrophoresis in 8 M urea-containing 15% polyacrylamide gels in 1X TBE buffer for 2 h. Fluorescently labelled DNA and DNA/RNA oligomers were detected by scanning using a Fujifilm FLA-5100 fluorescent image analyser.

### Ribonuclease and phosphatase assays

Ribonuclease and phosphatase assays were performed as described in (10). Briefly fluorescein-labeled DNA–RNA (30 nM) was incubated with 300 nM Mpa PE wt (or Mpa PE H82A) in 50 mM Tris (pH 7.5) and 5 mM MnCl2 at 37°C for the time indicated. The reactions were quenched by the addition of Stop buffer (95% [v/v] formamide, 0.09% [w/v] xylene cyanol) and the mixtures were boiled at 95°C for 10 min. Samples were separated by electrophoresis on an 8 M urea, 15% polyacrylamide gel in 1X TBE buffer for 2 h. Fluorescently labeled DNA/RNA oligomers were detected by scanning using a Fujifilm FLA- 5100 fluorescent image analyzer.

## RESULTS

### Crystal structure of an archaeal NHEJ phosphoesterase

**Figure 1.**
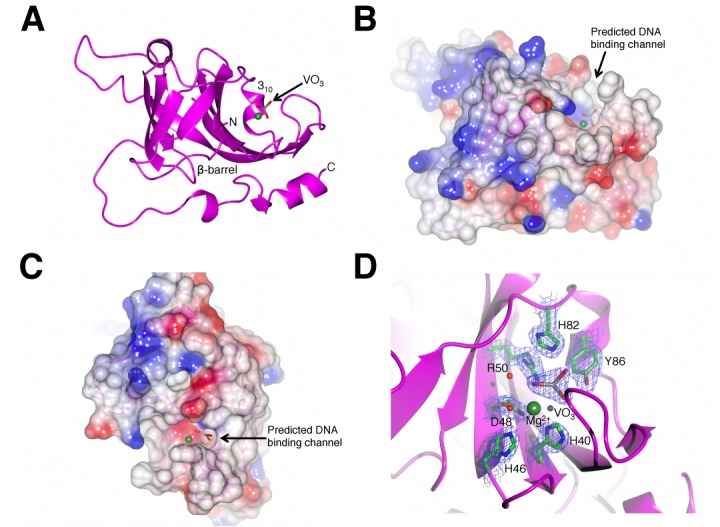
Crystal structure of an archaeal NHEJ phosphodiesterase. (**A**) A ribbon diagram representation of the crystal structure of *Mpa* PE. The hydrophobic core of the enzyme is comprised of a β-barrel containing 9 anti-parallel β-strands. A vanadate ion (grey) is bound in the active site adjacent to a 3_10_ helix. A bound magnesium ion is also indicated by a green sphere. (**B, C**) Surface representations of the *Mpa* PE crystal structure. The active site is denoted by the vanadate and magnesium ions, and the loop that encloses the active site is shown in a ‘closed’ position. (**D**) A ribbon diagram of the 1.79 Å resolution structure of *Mpa* PE with detail of the active site. H40, H46 and D48 are involved in coordinating a catalytic metal ion represented here by magnesium, whilst R50, H82 and Y86 are involved in coordinating the vanadate ion, which likely occupies the position of the scissile phosphate in phosphoesterase reactions. The side chains are covered by electron density maps (2*F*_o_-*F*_c_), shown in blue mesh with a 1.5-σ cutoff.

We previously reported that the *M. paludicola* phosphoesterase (*Mpa* PE), which is operonic with an NHEJ RNA/DNA primase–polymerase and ligase, is a functional component of the archaeal NHEJ repair apparatus. *Mpa* PE remediates DNA displacement synthesis by the NHEJ polymerase, enabling efficient ligation of broken DNA ends to restore the integrity of the genome. To understand more about the molecular mechanism of these atypical nucleases, we elucidated the crystal structure of *Mpa* PE at a resolution of 1.79 Å (Figure 1). A ribbon diagram of the PE protein shows a hydrophobic core formed of a β-barrel super secondary structure, composed of nine anti-parallel strands. The β-barrel is surrounded by four α-helices and a 3_10_ helix, as observed in a prokaryotic PE structure (Figure 1A) (21). The exterior of the β-barrel provides a cavity for the active site, which is enclosed by the 3_10_ helix and a surface loop. A surface representation of *Mpa* PE is shown in Figure 1B and C, in which vanadate and magnesium ions can be observed occupying a cleft. Since vanadate is a structural analogue for phosphate, we infer that the vanadate ion is occupying the position of the scissile phosphate, positioned in between the six key catalytic residues: H40, H46, D48, R50, H82 and Y86 (Figure 1D). We predict that this cleft is a DNA binding channel. The overall fold of the PE is unique to this clade of phosphoesterases, with no other 3′ ribonucleases or phosphatases having a similar catalytic site organization (21).

The unique PE fold appears highly conserved across prokaryotes and archaea, although it is unknown whether other archaeal or bacterial PEs participate in NHEJ. All existing PE crystal structures have a β-barrel core and a 3_10_ helix forming one side of the active site (compared in Supplementary Figure S1A–D, [21,32]). All three archaeal proteins have a surface loop opposite to the β-barrel, which forms the active site cleft, and it seems likely that the *Pae* PE also has this loop, although no electron density for this region was observed in the *Pae* structure, suggesting that it is highly flexible (21). The architecture of the structures implies that the surface loop is flexible and may enclose the DNA once the proper substrate is engaged for catalysis (Supplementary Figure S1E,1F). This speculation is supported by NMR solution studies that suggest that the crystal structures represents a ‘closed’ active site formation, and that the loop moves away from the active site in the absence of a substrate (33). This study also showed that upon addition of substrate, spectral perturbations were observed that indicated movement of the loop region, and formation of a ‘closed’ active site from an ‘open’ one.

### A conserved histidine is required for removal of annealed ribonucleosides and terminal 3′-phosphate

**Figure 2.**
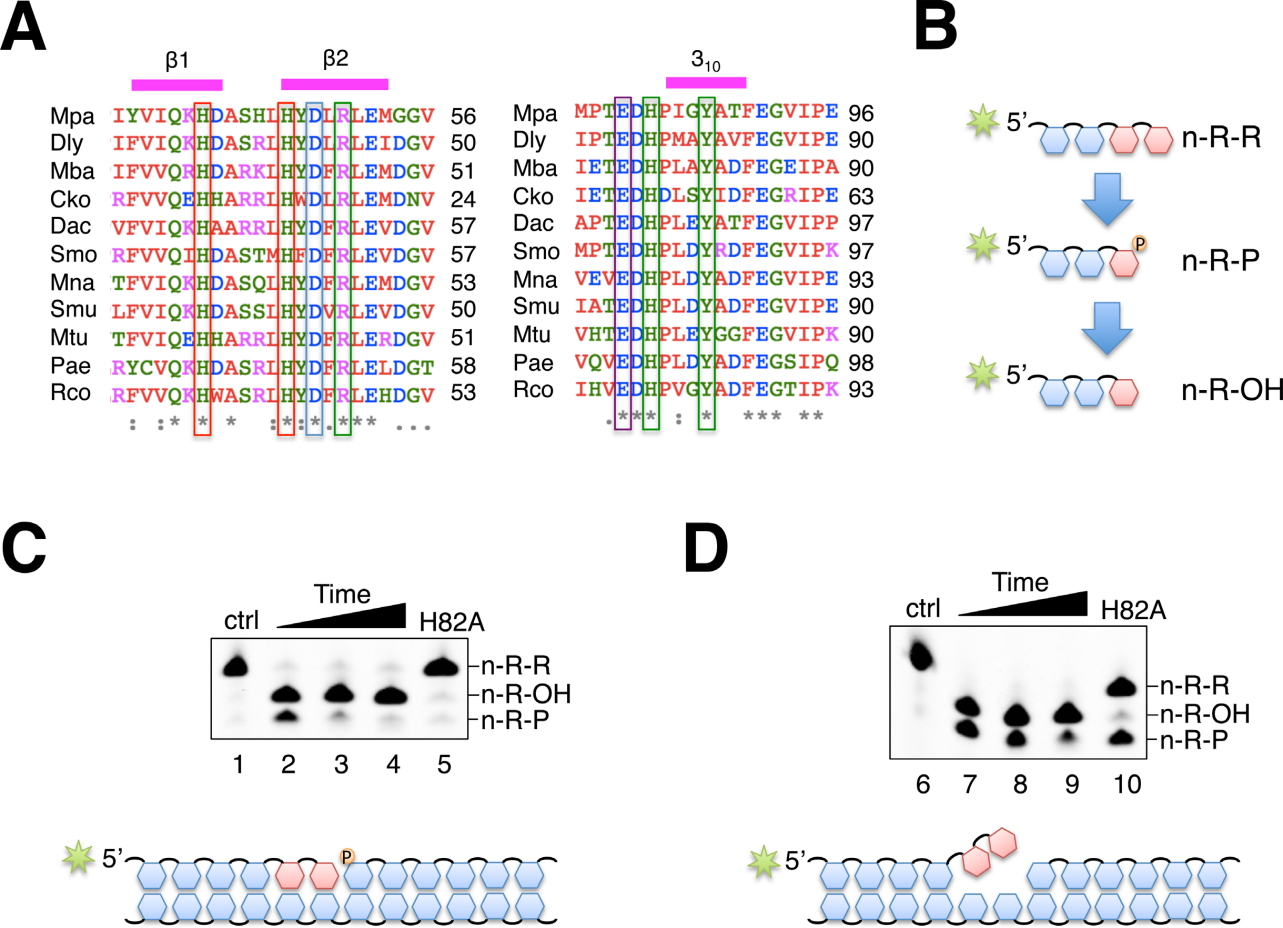
Critical function and conservation of active site residues in the AP–NHEJ phosphoesterase. (**A**) This amino acid alignment shows the three residues that coordinate the metal ion in the catalytic site (red box), whilst the three residues that coordinate the scissile phosphate are shown (green box). All six residues are strictly conserved in all species listed here. The conserved glutamate that is predicted to be essential for 3′-phosphatase activity is also shown (purple box). Aligned species; Mpa—*Methanocella paludicola* (archaea), Dly—*Dehalogenimonas lykanthroporepellens* (bacteria), Mba—*Methanosarcina barkeri* (archaea), Cko—*Candidatus korarchaeum cryptofilim* (archaea), Dac—*Desulfobacca acetoxidans* (bacteria), Smo—*Streptomyces monomycini* (bacteria), Mna—*Marinobacter nanhaiticus* (bacteria), Smu—*Salipiger mucosus* (bacteria), Mtu—*Mycobacterium tuberculosis* (bacteria), Pae—*Pseudomonas aeruginosa* (bacteria), Rco—*Ricinus Communis* (plantae). (**B**) A scheme showing the reaction mechanism for *Mpa* PE, detailing our nomenclature for the reaction products. n-R-R is a primer of indeterminate length that contains two successive ribonucleosides at the 3′ end. n-R-P is the intermediate reaction product following the removal of a ribonucleoside that still retains a 3′-phosphate. n-R-OH is the final reaction product that has had the 3′-phosphate moiety removed and terminates in a hydroxyl group. (**C**), (**D**) Phosphoesterase reactions contained 300 nM *Mpa* PE and 300 nM *Mpa* PE H82A mutant where indicated. Reactions also contained 30 nM 5′-fluorescein labelled substrate and 5mM Mn. The two 3′ RNA bases of substrate in (**D**) are non-complementary with the template strand, and create a 3′ flap. The wild type reactions were incubated for 30, 60 and 90 min, and the H82A mutants were incubated for 90 min, at 37°C.

The amino acid sequence of *Mpa* PE was used as a template to identify other potential AP–NHEJ PEs using PSI-BLAST. We aligned some known bacterial and archaeal PEs with several putative bacterial, archaeal, fungal and plant PEs that were identified during our search (Figure 2A, Supplementary Figure S2 for full alignment). All six key catalytic PE residues are strictly conserved across all species and the residues that comprise the β-strands of the hydrophobic core are also conserved. The N-terminal extension is missing in some species, thought to correlate with phosphoesterase activity (phosphate removal) (32,34). Notably, several species that have a truncated N-terminus also do not maintain the usually conserved glutamate residue implicated in phosphoesterase activity (Supplementary Figure S2). Six critical active site residues are highly conserved and are likely to be required to maintain phosphodiester cleavage activity specifically for AP–NHEJ repair. It is possible that species lacking the N-terminus region of PE may utilize another DNA phosphatase for removal of the 3′-phosphate.

The roles of a number of these catalytic residues in the PE active site, particularly those around the metal bindings sites, has been proposed based on mutagenesis and structural studies (34,35). However, the function of the more distal residues, such as the histidine, remains unclear. Analysis of the crystal structure of *Mpa* PE suggested that the conserved histidine residue (H82) may be one of the key residues in positioning the scissile phosphate in the active site (Figure 1D). To better understand the role of this histidine, we generated an alanine substitution mutant of H82 (PE^H82A^). Next, we assayed this mutant for its ability to perform phosphomono- and diesterase activities on DNA/RNA primers annealed to a DNA template. The reaction scheme is shown in Figure 2B. PE^H82A^ was completely unable to cleave the DNA/RNA substrate, in contrast to the wt *Mpa* PE control (Figure 2C). A similar DNA/RNA primer with two ribonucleosides non-complementary to the template DNA, essentially a short 3′ flap, was designed to examine the substrate specificity of *Mpa* PE (Figure 2D). Wt *Mpa* PE was less efficient at removing the 3′-phosphate from the flap than from an equivalent annealed substrate, but the phosphodiesterase activity was not reduced (Figure 2D). Strikingly, *Mpa* PE^H82A^ was able to remove the terminal ribonucleoside from this substrate, although less efficiently than wt, but was unable to remove the 3′-phosphate. These results establish that H82 is essential for 3′-phosphatase activity and also for topological manipulation of the annealed terminal nucleoside for phosphodiesterase activity. These data suggest that histidine 82 plays a key role in interrupting the base pairing between the incoming ‘primer strand’ nucleoside and the DNA template, allowing the correct positioning of the scissile phosphate in the active site. In the absence of H82, PE is unable to remove the annealed terminal nucleoside. However, the enzyme can remove a 3′ terminal nucleoside from a flap (unpaired DNA) in the absence of this histidine (Figure 2D). These findings also support the suggestion that the DNA phosphatase activity associated with PE occurs in the same active site as the ribonuclease activity. The previously discussed role of the N-terminal region of the PE on the DNA phosphatase activity remains unclear but we speculate that it may play a role in stabilizing substrate binding.

### Crystal structure of an archaeal NHEJ DNA polymerase

**Figure 3.**
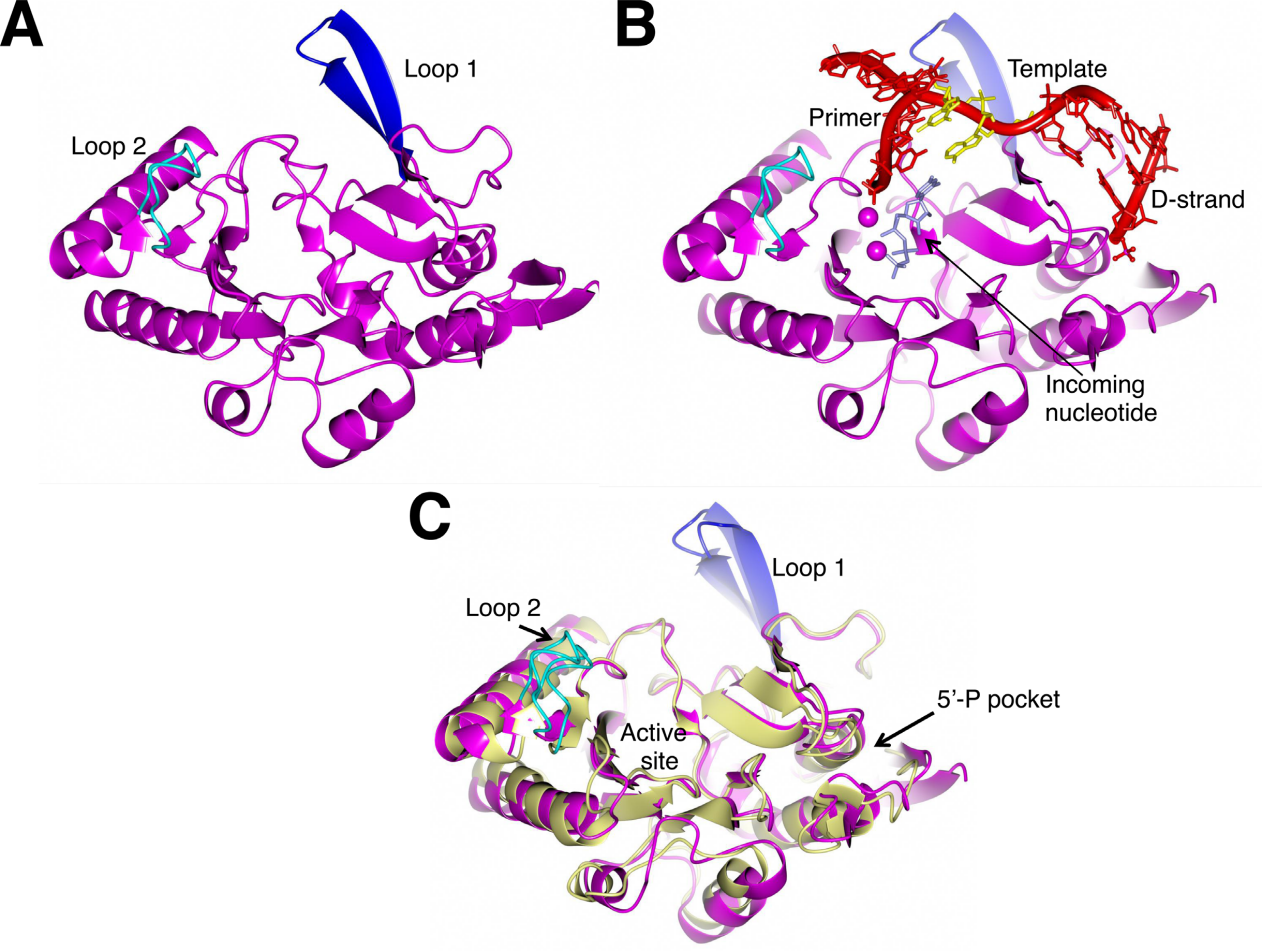
Structural and functional conservation of AP–NHEJ polymerases. (**A**) A ribbon representation of the crystal structure *Mpa* Pol. Loop 1 is highlighted in dark blue, whilst Loop 2 is highlighted in cyan. (**B**) A ribbon representation of the *Mpa* Pol crystal structure manually docked with an incoming nucleotide and DNA substrate derived from the *Mtu* PolDom structures PDB:3PKY and PDB:4MKY, respectively (15,36). (**C**) A comparison of loop positioning between *Mpa* Pol (magenta) and *Mtu* PolDom (cream). Loop 1 and Loop 2 are shown in dark blue and cyan, respectively. The loops occupy very similar positions, despite differing in amino acid composition, suggesting that the function of the loops is conserved. The *Mtu* PolDom figure was generated using the polymerase structure, PDB 2IRU (20).

To expand our structural understanding of other components of archaeal NHEJ apparatus, we sought to compare the structures of the bacterial NHEJ polymerases with that of the recently discovered archaeal Pol (9). We purified *Mpa* Pol and crystallized the enzyme, as described in the methods. The structure of *Mpa* Pol was elucidated at 1.95 Å resolution and a ribbon representation of its structural features is shown in Figure 3A. *Mpa* Pol shares close structural homology with the bacterial NHEJ polymerases from *M. tuberculosis* and *P. aeruginosa* (20,36). Based on the close structural similarities between all these polymerases, we can infer that the key structural elements such as Loop 1, Loop 2, the 5′-phosphate binding pocket and the active site (Supplementary Figure S3A, B) perform similar functions in the DSB repair process. Having shown that *Mpa* Pol is a *bona fide* member of the AP–NHEJ polymerase family, we next superposed the structure onto that of *Mtu* PolDom in complex with an incoming nucleotide and DNA (10,16,37). We found that the nucleotide and DNA substrate of the *Mtu* PolDom structures overlaid precisely with the *Mpa* Pol structure (Figure 3B). Figure 3C shows a superposition of *Mpa* Pol and *Mtu* PolDom with the surface loops 1 and 2 occupying highly similar positions. The *Mpa* and *Mtu* Pols do not have strictly conserved primary amino acid sequences, yet both Loop 1 and Loop 2 adopt similar formations, suggesting a conserved function. Together, these studies confirm that the archaeal and bacterial NHEJ polymerases are also structurally equivalent and likely function in similar ways during break repair.

### Structural elements of NHEJ Pol involved in displacement synthesis

**Figure 4.**
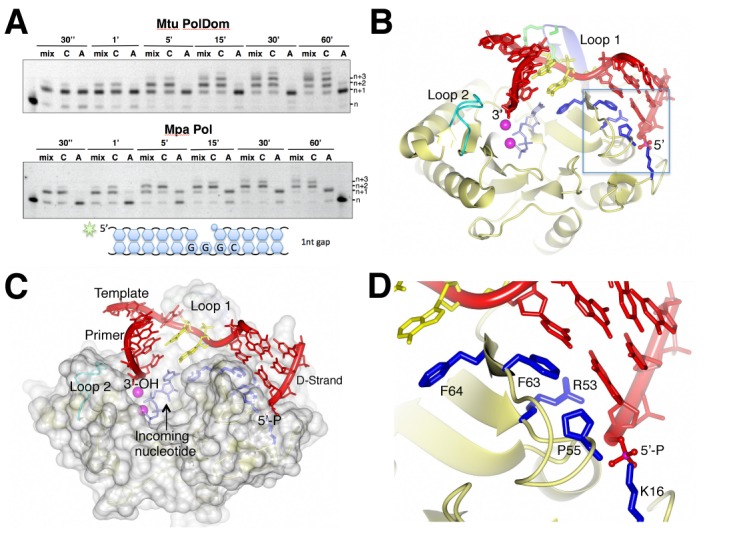
Structural features of NHEJ polymerases involved in displacement synthesis. (**A**) Gap filling extensions reactions contained 300 nM *Mtu* PolDom or *Mpa* Pol and 30 nM 5′-fluorescein labelled substrate. Reactions also contained 62.5 μM of either ATP, CTP, or a mixture of all 4 NTPs (mix), and 5 mM Mn and were incubated for the indicated time periods. (**B**) A ribbon representation of the crystal structure of *Mtu* PolDom engaging a 1 nucleotide gap substrate with an incoming nucleotide, generated with the previously published *Mtu* PolDom structures PDB:3PKY and PDB:4MKY (Brissett *et al*., 2011, 2013). The phenyalanines, arginine and proline residues that contact the downstream dsDNA interface are shown in stick format (dark blue). (**C**) A surface representation of the *Mtu* PolDom model with gap DNA substrate. The structural wedge that meets the downstream dsDNA is clearly visible in this format. The DNA substrate components are labelled as primer, template and D-strand (downstream strand). (**D**) A detailed view of dsDNA interface with *Mtu* PolDom and the potential amino acid residues involved in displacement synthesis, shown in ribbon and stick format.

As well as sharing common structural features, *Mtu* PolDom and *Mpa* Pol also share similar extension activities required for NHEJ-mediated break repair (10,14,20,36). We previously reported that *Mpa* Pol, in common with other NHEJ polymerases, is preferentially a DNA-dependent RNA polymerase with an innate capacity to perform strand displacement synthesis (10). To determine if mycobacterial NHEJ polymerases also possess this unusual activity, we compared the displacement synthesis activity of *Mpa* Pol with *Mtu* PolDom on gapped DNA substrates. When incubated with a DNA substrate with a single nucleotide gap over an increasing time course, both *Mtu* and *Mpa* Pols displaced the downstream strand (D-strand) during RNA synthesis to incorporate up to 3 or 4 bases (Figure 4A). This establishes that gap-filling displacement synthesis is also present in the mycobacterial enzymes, and is likely conserved across the NHEJ polymerase family. Displacement synthesis is a potentially dangerous activity to possess, requiring PE-mediated resection to keep it in check, therefore this suggests that this activity is maintained to provide some important role in DNA break repair that remains to be identified.

To clarify the molecular basis for displacement synthesis, we examined the crystal structures of *Mtu* PolDom in complex with DNA to identify if structural elements of NHEJ Pols are involved in strand displacement activity (16,37). By combining a pre-ternary structure (Pol, D-strand, template, incoming nucleotide; PDB: 3PKY) with a catalytically competent MMEJ synaptic structure (Pol, D-strand, template, primer; PDB: 4MKY), we obtained a representative structure of an AP–NHEJ Pol engaging a single nucleotide gap substrate in preparation for nucleotide incorporation (Figure 4B and C). Figure 4B highlights the key structural elements of the Pol (Loop1, Loop 2 and 5′-P binding pocket), whilst the surface representation in Figure 4C demonstrates the broad catalytic cleft and the significant architecture that serves to hold the 5′-P end of the gap and to splay the template open to allow for base-pairing with an incoming nucleotide. Closer examination of this structure reveals a prominent surface ‘wedge’ formed by five residues (K16, R53, P55, F63 and F64) that make contacts with or near the annealed dsDNA interface. A ribbon representation of this molecular wedge is shown in Figure 4D. As previously reported, the phenylalanines play critical roles in ‘opening’ the DNA template, whilst the lysine is essential for binding to the 5′-P (15,37). The importance of R53 and P55 in DNA manipulation has not previously been described. Given their prominence in the region of strand-displacement, we chose to mutate these five residues (K16, F63, F64, R53, P55) in *Mtu* PolDom in order to characterize their potential roles in strand displacement synthesis.

### 5′ phosphate docking site is not required for strand displacement

**Figure 5.**
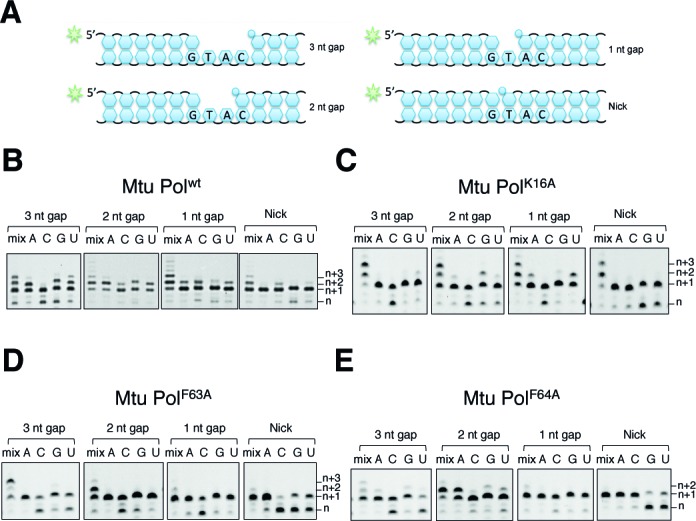
A comparison of dislocation activities of residues potentially involved in displacement synthesis. (**A**) A schematic of the gapped DNA substrates used for the gap filling and displacement synthesis reactions, including details of the templating bases in the gap. (**B-E**) DNA extension assays with *Mtu* PolDom wt, K16A, F63A and F64A, respectively. Reactions contained 300 nM AP–NHEJ polymerase with 30 nM 5′-fluorescein labelled substrate and 5mM Mn and were incubated for 1 h at 37ºC. Reactions contained either a mix of NTPs or individual NTPs (ATP, CTP, GTP or UTP) as indicated.

Previously, we reported that lysine 16 in *Mtu* PolDom (K19 in *Mpa* Pol) hydrogen bonds with the 5′-phosphate (5′-P) of the downstream DNA strand (D-strand) (Supplementary Figure S3B) (15). This docking facilitates stable binding of the polymerase to DNA and formation of a pre-ternary complex (Pol, D-strand, incoming nucleotide), which can assemble in the absence of an incoming primer strand (16). Our previous work has demonstrated that an AP–NHEJ polymerase can displace the D-strand during gap-filling synthesis (10). We hypothesized that loss of K16 might create increased mobility of the D-strand and therefore prevent displacement synthesis. To investigate the potential role of this residue in displacement synthesis during gap filling, this lysine was mutated to alanine (PolDom^K16A^). We then compared the displacement activities of wt PolDom and PolDom^K16A^ on four different DNA substrates (nicked, 1, 2 or 3 nucleotide gap) (Figure 5A, B and C). The extension activity of PolDom^K16A^ was comparable to wt PolDom, with both enzymes able to fill in gaps on all substrates and also perform displacement synthesis with the nicked, 1 nt and 2 nt gapped substrates. These results indicate that K16 is not critical for displacement synthesis and does not play a direct role in ‘opening’ the downstream DNA. Both wild type and mutant enzymes showed low fidelity DNA-directed RNA synthesis, as each was proficient in incorporating mis-paired bases.

### Loss of hydrophobic base stacking residues reduces gap filling and strand displacement activities

As previously reported, loss of the prominent base stacking residues F63 and F64 significantly reduces, but does not ablate, strand synthesis (Figure 5D and E) (37). PolDom^F63A^ and PolDom^F64A^ mutants are capable of filling in 1 nt and 2 nt gaps but both mutants showed reduced displacement synthesis compared to wt Pol, especially on the nicked substrate. However, neither of these residues appears critical to displacement synthesis, since the activity still occurs to some degree in their absence, even on DNA nick substrates. Given the combined template-splaying activity of these phenylalanine residues, it is difficult to unpick their role in DNA extension from a potential role in displacement synthesis. We conclude that F63 and F64 may encourage the separation of the downstream dsDNA but are not essential for the process since it still occurs in a limited manner after their substitution to alanine.

### Arginine 53 is critical for strand displacement synthesis

**Figure 6.**
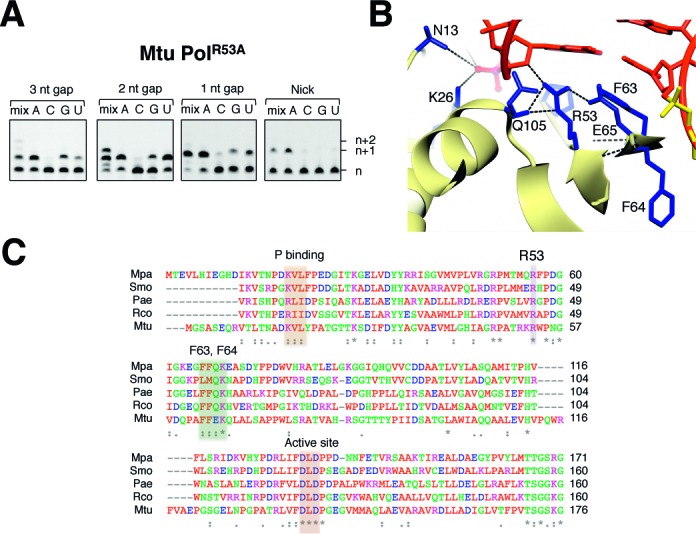
R53 is directly involved in splaying the template/D-strand junction of DNA to allow displacement synthesis to occur. (**A**) A DNA extension assay containing 300 nM *Mtu* PolDom^R53A^, with 30 nM 5′-fluorescein labelled substrate and 5mM Mn. Reactions contained either a mix of NTPs or individual ATP, CTP, GTP or UTP, as indicated, and reactions were incubated for 1 h at 37ºC. (**B**) The binding contacts made by R53 of *Mtu* PolDom with neighbouring residues and the nucleotides at the ss/ds DNA interface (**C**) The conserved phosphate binding pocket is highlighted in orange, the strictly conserved arginine implicated in displacement synthesis is highlighted in purple, the DNA splaying phenylalanine residues are shown in green, and the conserved active site residues are shown in red. These key regions of the AP–NHEJ are conserved across bacteria, archaea and even plants. Aligned species; Mpa—*Methanocella paludicola* (archaea), Smo—*Streptomyces monomycini* (bacteria), Pae—*Pseudomonas aeruginosa* (bacteria), Rco—*Ricinus Communis* (plantae), Mtu—*Mycobacterium tuberculosis* (bacteria).

Arginine 53 and proline 55 both directly contact the annealed dsDNA as it transitions to ssDNA. We anticipated that alanine substitution of either residue might ablate strand displacement synthesis, however we observed that loss of the proline (PolDom^P55A^) had little impact on the activity (Supplementary Figure S4), whilst loss of the arginine (PolDom^R53A^) produced the most striking results of all the mutants with all three gapped and nicked DNA substrates (Figure 6A). Although PolDom^R53A^ was catalytically active and able to fill in 1 and 2 nucleotide gaps, when supplied with a mixture of NTPs in the case of the former, it had completely lost strand displacement synthesis activity associated with the wt enzyme. Multiple nucleotides can be added (2 nt gap substrate) but R53A did not add any additional nucleotides to ‘over-fill’ gapped DNA, and it was unable to insert any nucleotides in the nicked DNA substrate. Together, these results establish that R53 plays a pivotal role in strand displacement synthesis, likely acting to disrupt the annealed downstream DNA strand. Examination of the PolDom–DNA complex structure revealed that R53 intercalates into the annealed DNA interface, making hydrogen bond contact with the ribose of the ultimate base of the D-strand (Figure 6B). It appears that this arginine serves as a wedge to open the DNA ‘zipper’, thus facilitating the disruption and mobility of both the downstream and template strands. A sequence alignment of the bacterial, archaeal and plant AP–NHEJ Pols demonstrates that this arginine is strictly conserved, along with the key catalytic aspartate residues (Figure 6C). We propose that this residue has been maintained as a feature of functional importance in break repair. The likely roles of this conserved polymerase displacement synthesis activity in DSB repair is discussed below.

## DISCUSSION

When repairing discontinuous DSBs with a limited toolbox, the chances of success are greatly increased if the available tools are either multifunctional or flexible enough to bind to and modify a diverse range of different substrates. The AP–NHEJ repair apparatus epitomizes these principles, having evolved a highly adaptable set of enzymes that together can handle and manipulate the varied DSB ends it encounters, in preparation for the final step of break rejoining. NHEJ polymerases assist in bringing discontinuous DNA termini together and then fill in resulting gaps or incorporate nucleotides at blunt ends to promote MMEJ. However, these polymerases tend to over fill gaps, which may aid in swift repair. PE can resect unnecessarily lengthened stretches of newly incorporated RNA and can also remove 3′-phosphates from DNA or RNA termini to allow the generation of a 3′ hydroxyl moiety that is requisite for DNA synthesis and ligation. Finally, the ligase seals the nicks once they have been appropriately configured for resealing.

Crystallographic studies reported here on the only archaeal PE directly implicated in NHEJ repair processes, demonstrates their close homology with the equivalent bacterial phosphoesterases. Both are highly structurally conserved and share a broad catalytic cleft with a flexible loop, which has been proposed to enclose the DNA/RNA substrate (21). Loss of a conserved histidine in the active site of PE prevents the enzyme from being able to resect annealed substrates, however it remains competent in resecting flap substrates. We interpret these data to indicate the role of the histidine in disrupting the base pairing between the terminal ribonucleoside and the DNA template thus enabling the scissile phosphate to occupy the optimum location in the active site for cleavage. We previously showed that the PE is capable of resecting RNA from a DNA substrate with overhanging 3′-DNA flaps; viewed alongside these latest results it reinforces PE's remarkable ability to manipulate DNA/RNA substrates following displacement synthesis and provides novel insights into how this might be achieved. The next crucial step must be to obtain a co-crystal ternary structure of PE bound to an appropriate DNA–RNA substrate that will provide a molecular understanding of how these nucleases resect strand-displaced intermediates.

AP–NHEJ polymerases possess distinctive DNA binding mechanisms that allow them to operate even at the extreme termini of DSBs. A positively charged surface pocket binds specifically to 5′ phosphates thus stably anchoring the enzyme to DNA to permit efficient end-processing. Prominent surface loops (Loops 1 and 2) enable these polymerases to directly promote break synapsis by MMEJ. During this break annealing process, each side of the DSB is bound by a polymerase and the surface loops promote break synapsis (14). In the case of the 3′ overhangs, this mechanism configures the DSB to facilitate gap-filling synthesis *in trans* (16,37). The structural architecture of the archaeal NHEJ Pol reported here shows that it is highly similar to the bacterial NHEJ polymerases, supporting conservation in the functional mechanisms of these enzymes in DSB repair.

Both bacterial and archaeal NHEJ polymerases readily perform strand displacement synthesis, when operating on ‘gapped’ DNA substrates possessing a downstream strand. A conserved arginine residue (R53 in *Mtu* Pol), that contacts the annealed juncture of 5′-DNA end, plays a critical role in enabling the local unwinding of the DNA helix. This forms part of a molecular wedge that allows displacement synthesis activity to occur, resulting in ribonucleotide incorporation into the previously annealed duplex. An analogous process appears to be utilized by other polymerases to promote base unpairing (38,39). A structure of the *Thermococcus gorgonarius* family B DNA polymerase (Tgo PolB) revealed that an arginine residue contacts the primer/template junction in a similar way to R53 in *Mtu* PolDom and, together with a neighbouring tyrosine side chain, disrupts base stacking on the primer strand. This allows strand displacement to occur that facilitates subsequent removal of misincorporated bases by the 3′ exonuclease activity of this replicative polymerase thus promoting proofreading. In the case of the AP–NHEJ polymerases, the arginine residue physically promotes separation of the dsDNA to allow for displacement synthesis activity.

**Figure 7.**
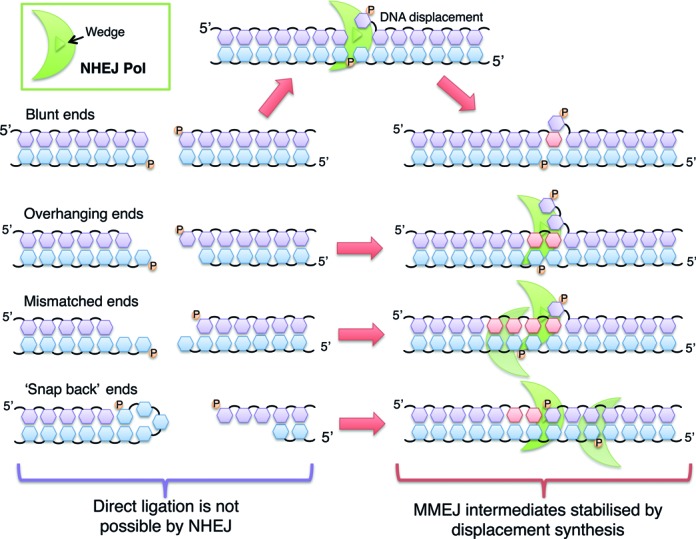
Potential roles for displacement synthesis during NHEJ repair. A schematic diagram demonstrating how NHEJ polymerases (green) may utilise displacement synthesis in order to temporarily stabilise DSB intermediates. NHEJ Pols can ingress into fully annealed blunt DNA ends or overhanging ends. The arginine-tipped wedge in the polymerase allows the annealed DNA to be ‘opened’ to expose regions of microhomology. This MMEJ process enables the break termini to be synapsed back together, forming more stable intermediates that can be further processed and repaired more efficiently and precisely. This displacement synthesis mechanism can be likened to the joining of the ends of a broken zip, with the polymerase acting as the slider that adds teeth as it moves across the break to gain traction with the other side. The new teeth displace the ones that were already zipped, allowing the two broken ends to become more stably connected. After this connection has been made, the synapsed break can then be rejoined back together.

The structures of these NHEJ polymerases bound to DNA, combined with biochemical evidence, demonstrates that strand displacement activity is not an artifact but results from the presence of a prominently conserved wedge structure that induces a significant distortion in the template strand DNA that it is an intrinsic part of its synthesis mechanism. However, the exact requirement of this conserved displacement activity in DSB repair is not immediately obvious and, in fact, is a potentially dangerous activity to possess. One possibility is that displacement synthesis increases flexibility in DSB repair by increasing the variety of reconfiguration options to suit the potentially huge array of mismatched or damaged DNA ends that may occur following a break (Figure 7). Displacement synthesis may also allow for generation of microhomologies at blunt-ended DSBs. Significantly in this regard, it has been directly observed that these polymerases can ingress into blunt ends (16), potentially uncovering regions of ssDNA that can promote MMEJ with the adjacent break terminus. Another possibility is that ssDNA overhangs have a tendency to ‘snapback’ and anneal to the same strand, forming hairpin structures that are potentially refractory to processing and ligation thus preventing efficient end-modification, MMEJ and, ultimately, inhibiting DSB repair. Strand displacement may act as a molecular ‘plough’ that displaces such intermediates to minimize such aberrant annealing thus promoting more efficient MMEJ and break repair.

However, although these possibilities are feasible and may partially account for the necessity to perform displacement synthesis, they do not adequately explain the strong tendency of these polymerases to ‘over fill’ gaps at MMEJ-synapsed termini. A crucial step in polymerase-mediated MMEJ process is the formation of stably synapsed DNA intermediates. This is not an energetically unfavourable task when DSB overhangs are long and complementary and probably require minimal intervention to promote break annealing. However, MMEJ can occur using homologies as little as 1–2 base pairs and, in such scenarios, these intermediates can readily unanneal if this ‘foothold’ is not reinforced. It is likely that strand displacement synthesis plays an important role in stabilizing such unstable MMEJ intermediates. This process can be likened to the procedure of bringing two sides of a zipper back together (Figure 7). The first step in this process is to engage a few teeth with the other side of the zipper to make an initial connection and alignment. However, at this stage, the teeth can easily disengage unless the process can be rapidly advanced to create more traction so that then the zipper becomes stably engaged. Analogously, once the polymerase has promoted synapsis of short microhomologies, it actively extends these intermediates in order to add more ‘teeth’ (nucleotides) than are necessary to ‘encourage’ the unstable frayed ends of the break to remain annealed. Displacement synthesis therefore provides a mechanism to reinforce greater stability to an initially unstable synaptic intermediate formed during the MMEJ process. Critically, by displacing the downstream strand with more ribonucleotides, the ends of the break can become more stably associated. This stable connection then allows the other strand of the DSB to also become stably engaged followed by further processing by PE to remove excess RNA and configure the break for subsequent ligation. This also possibly explains why RNA is used as a repair intermediate, as these temporary ‘teeth’ are demarcated as newly added bases that are subsequently resected and replaced with deoxyribonucleotides (10), either via reannealing of the displaced strand or via resynthesis by more accurate DNA patch repair polymerases.

It has recently been reported that Pol θ is required for MMEJ repair in mammalian cells (40). Human Pol θ, in common with the AP–NHEJ Pols, also appear to have the capacity to promote DSB synapsis by MMEJ followed by DNA synthesis to fill in any remaining gaps. Notably, Pol θ can also displace annealed downstream strands. Together, these findings suggest that an analogous polymerase-mediated MMEJ mechanism is also employed to repair non-homologous DSBs in mammalian cells. It has been proposed that during this MMEJ mechanism, resulting 5′-flaps would be resected to prepare the DNA for ligation, in contrast to AP–NHEJ that appears to favour 3′-end resection by PE. However, it remains a distinct possibility that some kind of equivalent PE-like resection process also exists in eukaryotic cells.

## ACCESSION NUMBERS

Atomic coordinates and structure factors have been deposited in the Protein Data Bank with accession codes 5DMP and 5DMU for the *Mpa* phosphoesterase and *Mpa* polymerase domains, respectively.

## Supplementary Material

SUPPLEMENTARY DATA
