# Supplementary material for: Molecular basis for DNA strand displacement by NHEJ repair polymerases
Source: Nucleic Acids Res. 2015 Sep 23;44(5):2173–86. doi: 10.1093/nar/gkv965 (PMC4797286; doi:10.1093/nar/gkv965)
Supplement: SUPPLEMENTARY DATA [file supp_44_5_2173__index.html]

Molecular basis for DNA strand displacement by NHEJ repair polymerases — SUPPLEMENTARY DATA 

# Molecular basis for DNA strand displacement by NHEJ repair polymerases

## SUPPLEMENTARY DATA

- SUPPLEMENTARY DATA
