# Supplementary material for: Molecular basis for DNA strand displacement by NHEJ repair polymerases
Source: Nucleic Acids Res. 2015 Sep 23;44(5):2173–86. doi: 10.1093/nar/gkv965 (PMC4797286; doi:10.1093/nar/gkv965)
Supplement: SUPPLEMENTARY DATA [file supp_gkv965_nar-01967-d-2015-File011.pdf]

Mutation	Primer
Mtu PolDom K16A	Fwd- See Reference 20.
	Rev- See Reference 20.
Mtu PolDom R53A	Fwd- See reference 20.
	Rev- See reference 20.
Mtu PolDom P55A	Fwd- 5'-CGC TGG GCG AAC GGC GTC GAC CAA CCC G-3'
	Rev- 5'-CGA CGC CGT TCG CCC AGC GCT TGC GCG TG-3'
Mtu PolDom F63A	Fwd- 5'-CCC GCG GCC TTC GAA AAG CAG TTG GCG-3'
	Rev- 5'-GCT TTT CGA AGG CCG CGG GTT GGT CGA C-3'
Mtu PolDom F64A	Fwd- 5'-GAC CAA CCC GCG TTC GCG GAA AAG CAG TTG GCG TTG TCG-3'
	Rev- 5'-CGA CAA CGC CAA CTG CTT TTC CGC GAA CGC GGG TTG GTC-3'
Mpa PE H82A	Fwd- 5'-GGC CAT GCC CAC GGA GGA TGC TCC CAT CGG CTA CGC CAC C-3'
	Rev- 5'-GGT GGC GTA GCC GAT GGG AGC ATC CTC CGT GGG CAT GGC C-3'

Table S1. A list of primers used for site-directed mutagenesis of Mtu PolDom and Mpa PE.

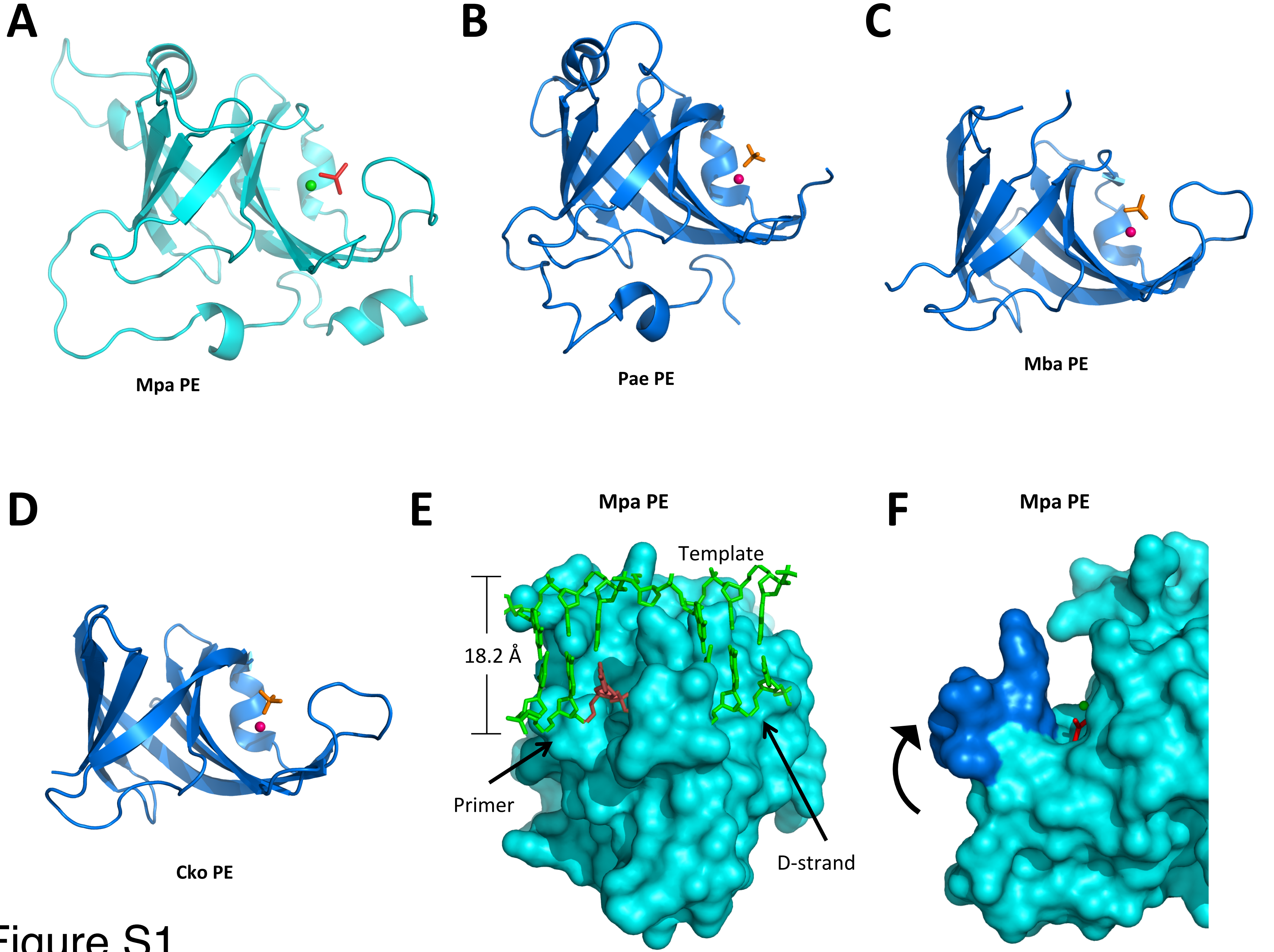
A
Mpa PE
B
Pae PE
C
Mba PE
D
Cko PE
E
Mpa PE
Template
18.2 Å
Primer
D-strand
F
Mpa PE

Figure S1

Figure S1. A comparison of crystal structures of four AP PEs, along with DNA/RNA binding models.

(A-D) Ribbon representations of the crystal structures of phosphoesterases from *Methanocella paludicola* (Mpa), *Pseudomonas aeruginosa* (Pae), - *Methanosarcina barkeri* (Mba) and *Candidatus korarchaeum cryptofilim* (Cko), respectively (9, 20, 31). Magnesium is shown in green, manganese in pink, vanadate in red and sulfate in orange. The PDB accession codes for the Mpa, Pae, Mba and Cko PEs are: 5DMP, 3N9B, 3P43 and 3P4H, respectively. **(E)** A model of how DNA might dock with the Mpa PE structure with surface representation, showing the primer DNA/RNA strand entering the active site. **(F)** A view of the predicted DNA channel in Mpa PE with the "closed" loop region coloured in blue to indicate the area that might enclose on the DNA/RNA substrate**.**

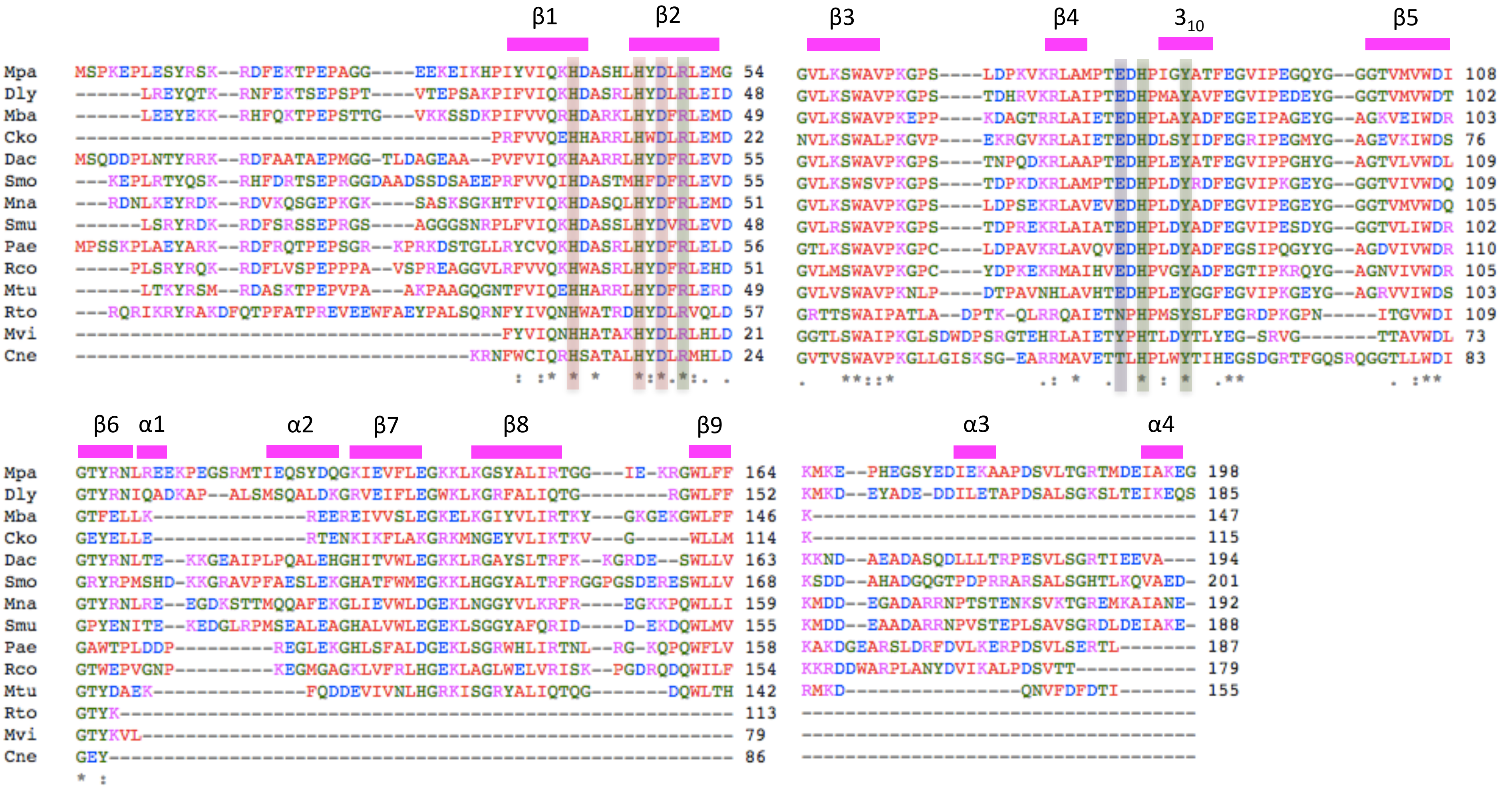

Figure S2. Alignment of the amino acid sequence of Mpa PE with related phosphoesterases

The overall alignment shows that the regions the comprise the β-strands of the β-barrel in Mpa PE are broadly well conserved, along with the 3_{10} helix. The short loop regions and short α-helical segments are not as strictly conserved. The three residues that coordinate the metal ion in the catalytic site are highlighted in red, whilst the three residues that coordinate the scissile phosphate are shown highlighted in green. All six residues are strictly conserved in all species listed here. The glutamate that is predicted to be essential for 3'-phosphatase activity is highlighted in purple, and is conserved in all but three species. These species appear to maintain truncated forms of the PE, which may no possess the DNA phosphatase activity along with the phosphodiesterase activity. Aligned species; Mpa – *Methanocella paludicola* (archaea), Dly - *Dehalogenimonas lykanthroporepellens* (bacteria), Mba - *Methanosarcina barkeri* (archaea), Cko – *Candidatus korarchaeum cryptofilim* (archaea), Dac - *Desulfobacca acetoxidans* (bacteria), Smo - *Streptomyces monomycini* (bacteria), Mna – *Marinobacter nanhaiticus* (bacteria), Smu – *Salipiger mucosus* (bacteria), Pae – *Pseudomonas aeruginosa* (bacteria), Rco – *Ricinus Communis* (plantae) , Mtu – *Mycobacterium tuberculosis* (bacteria), Mvi – *Microbotryum violaceum p1A1 Lamole* (fungi), Cne – *Cryptococcus neoformans var. grubii H99* (fungi).

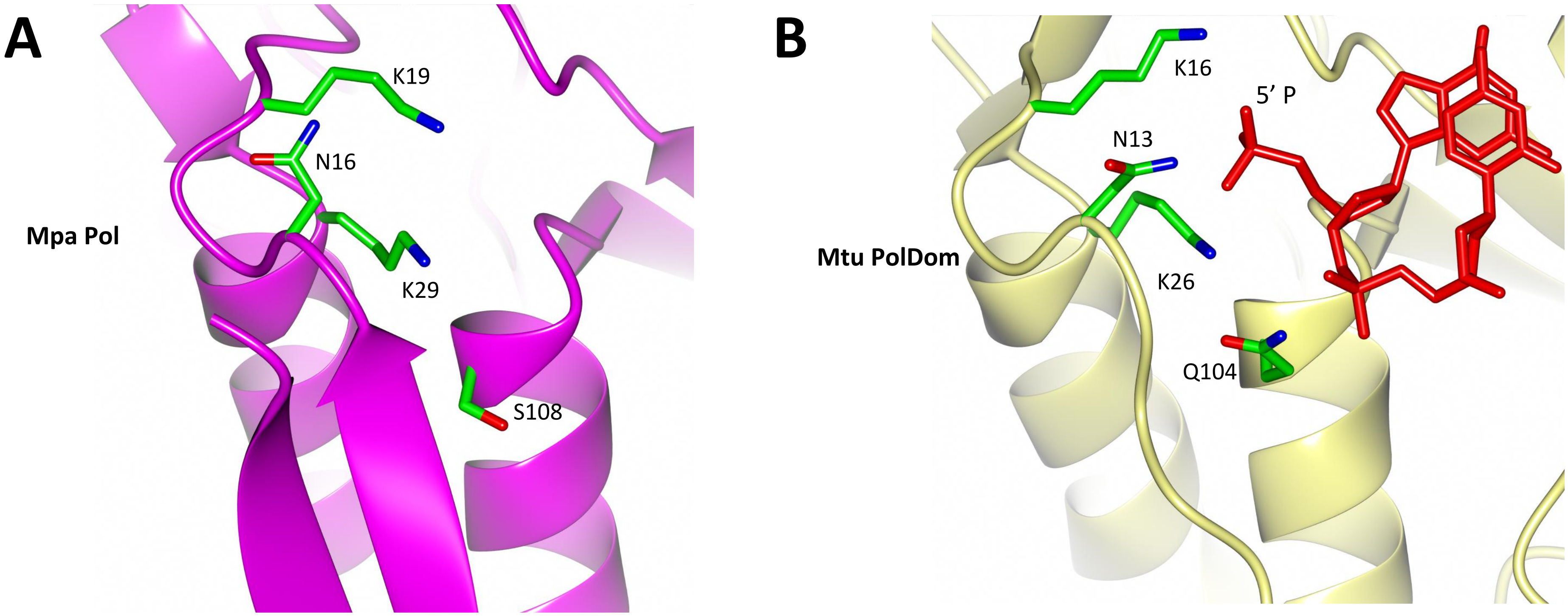

Figure S3. Comparing the 5’-phosphate binding sites in NHEJ Pol structures.
(**A**) A ribbon diagram of Mpa Pol, showing the phosphate binding pocket, composed of α1, α2, β1, and β2. The conserved 5’-P interacting residues are shown in blue (N36, K39, K49). S128 is in place of a conserved glutamine residue found in Mtu, Msm and Pae PolDoms. (**B**) A ribbon diagram of Mtu PolDom (PDB code: 3PKY) showing an incoming 5’-P interacting with conserved residues in blue (N13, K16, K26). The conserved glutamine (Q104) is shown in magenta, and is stabilising K26 by hydrogen bonding.

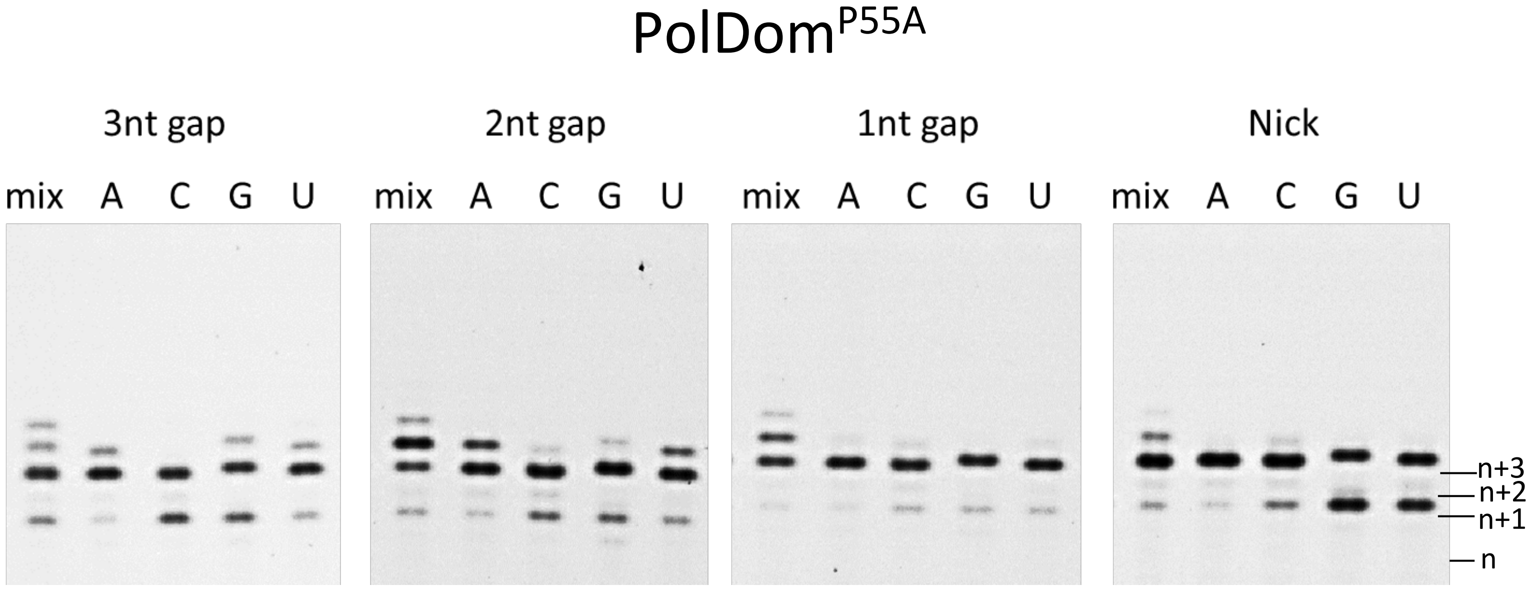

Figure S4. Strand displacement activity of Mtu PolDom^{P55A}

DNA extension assays with Mtu PolDom^{P55A}. Reactions contained 300 nM AP-NHEJ polymerase with 30 nM 5’-fluorescein labelled substrate and 5mM Mn and were incubated for 1 hour at 37ºC. Reactions contained either a mix of NTPs or individual ATP, CTP, GTP or UTP as indicated.
